# Unique transcriptomes of sensory and non-sensory neurons: insights from Splicing Regulatory States

**DOI:** 10.1038/s44320-024-00020-1

**Published:** 2024-03-04

**Authors:** Ludovica Ciampi, Luis Serrano, Manuel Irimia

**Affiliations:** 1 Center for Genomic Regulation, Barcelona Institute of Science and Technology, Barcelona, Spain; 2 Universitat Pompeu Fabra, Barcelona, Spain; 3 ICREA, Barcelona, Spain

**Keywords:** Regulatory States, Cell Types, Sensory Neurons, Photoreceptors, Alternative Splicing, Chromatin, Transcription & Genomics, Neuroscience, RNA Biology

## Abstract

Alternative Splicing (AS) programs serve as instructive signals of cell type specificity, particularly within the brain, which comprises dozens of molecularly and functionally distinct cell types. Among them, retinal photoreceptors stand out due to their unique transcriptome, making them a particularly well-suited system for studying how AS shapes cell type-specific molecular functions. Here, we use the Splicing Regulatory State (SRS) as a novel framework to discuss the splicing factors governing the unique AS pattern of photoreceptors, and how this pattern may aid in the specification of their highly specialized sensory cilia. In addition, we discuss how other sensory cells with ciliated structures, for which data is much scarcer, also rely on specific SRSs to implement a proteome specialized in the detection of sensory stimuli. By reviewing the general rules of cell type- and tissue-specific AS programs, firstly in the brain and subsequently in specialized sensory neurons, we propose a novel paradigm on how SRSs are established and how they can diversify. Finally, we illustrate how SRSs shape the outcome of mutations in splicing factors to produce cell type-specific phenotypes that can lead to various human diseases.

## Abstract (web summary)

This Review discusses the general rules of cell type- and tissue-specific alternative splicing programs in the brain and in specialized sensory neurons and proposes a new paradigm on how ‘splicing regulatory states’ are established, how they can diversify and how they are linked to human diseases.

## Graphical abstract

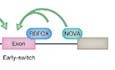

## Introduction

**Figure 1:**
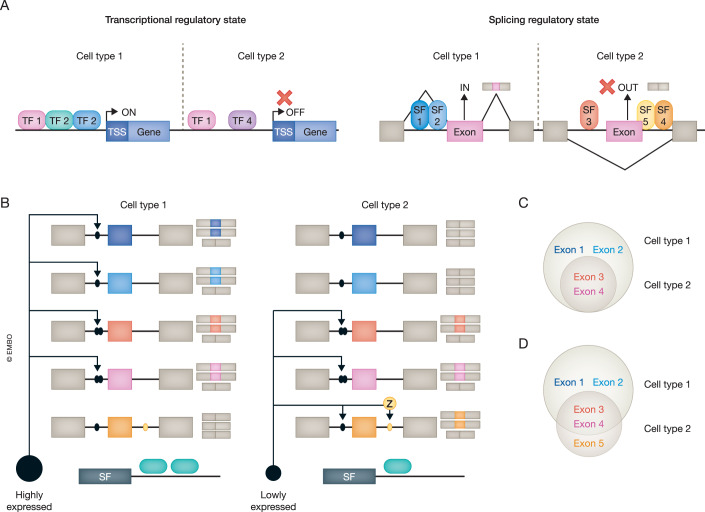
Transcriptional and splicing regulatory programs. (**A**) Schematic representation of the regulatory state of gene transcription (left) and AS (right). The regulatory state consists of transcription factors or splicing factors co-expressed in a given nucleus and whose combination defines gene expression/silencing or exon skipping/inclusion, respectively. TF transcription factor, SF splicing factor, TSS transcription start site. (**B**) In Cell type1, where a SF is highly expressed, most target exons become included at high levels, configuring a specific program for Cell type 1. In Cell type 2, where the SF is expressed at a lower, but substantial, level, only the most sensitive targets of the Cell type1 program become significantly included (exons 3 and 4; whose higher sensitivity is depicted by two ellipses as binding motifs). (**C**) These scenarios result in nested specific programs for Cell type 1 and 2. (**D**) If auxiliary (extrinsic) factors (Z, in yellow in panel **B**) are needed for recognition of some exons by the SF (e.g. exon 5), this will in turn favor non-overlapping programs.

Understanding how a single genome sequence can give rise to the extensive variety of cell and tissue types that compose a complex multicellular organism is fundamental challenge in biology. Decades of research in cell and developmental biology have shown that this is achieved by tight transcriptional and post-transcriptional regulation during embryogenesis and adulthood. At the transcriptional level, regulation is largely achieved by the interplay between transcription factors (TFs) and chromatin remodelers. Davidson and colleagues defined the (Transcriptional) Regulatory State of a given cell as “the set of functionally active transcription factors expressed together within a nucleus at levels high enough to occupy relevant DNA-binding sites and execute regulatory function” (Peter and Davidson, 2016; Peter, 2017; Davidson, 2006) (Fig. 1A).

At the post-transcriptional level, multiple cross-talking mechanisms diversify cell type-specific transcriptomes, modulating the identity, stability, localization, and translation of transcripts generated from a limited gene complement. Among them, Alternative Splicing (AS), the differential processing of introns and exons to produce various isoforms per primary transcript, is a major contributor to this diversification in animals. Similar to the Transcriptional Regulatory States, we can envision that AS regulation is achieved through the combined action of Splicing Factors (SFs) acting in a cell’s nucleus, constituting its Splicing Regulatory State (SRS) (Fig. 1A). Each SRS will therefore give rise to a distinct AS outcome, i.e. a set of gene isoforms expressed at certain ratios.

Here, we utilize the SRS framework to discuss three main topics. Firstly, we review the general principles governing cell type- and tissue-specific AS in the central nervous system (CNS), showcasing the example of neural microexon regulation. Through this exploration, we aim to establish a fundamental and integrative understanding of the broad neural SRS. Secondly, we focus on the unique AS repertoire observed in retinal photoreceptors, highlighting their differences compared to other neurons, and how this repertoire emerges from a distinctive SRS. Finally, we extend our focus to other sensory neurons, discussing the morphological and functional characteristics of sensory ciliated structures and speculating on how unique AS programs contribute to these features.

## Target architecture of cell type-specific regulators and the “nested programs” model

Irrespective of the regulatory layer (transcription or splicing), components of the corresponding Regulatory States share much of their molecular logic (Mantica and Irimia, 2022). For instance, we can broadly distinguish two types of regulators: (i) general factors, which act broadly across cell and tissue types, and (ii) cell type- and tissue-specific (CTS) factors, which are active only in one or few cell or tissue types. On one hand, general factors include core components of the basic cellular machineries (e.g. RNA polymerase complexes or the spliceosome), as well as other broadly expressed proteins that establish complex synergistic and antagonistic interactions to enhance or inhibit their targets (e.g. SR protein and hnRNPs in the case of splicing (Fu and Ares, 2014; Huelga et al, 2012)). On the other hand, CTS factors play particularly important roles in defining Regulatory States by strongly contributing to generate unique transcriptomes. Independently of the regulatory layer that they control, CTS factors are normally characterized by: (i) showing substantial expression in only one or few cell or tissue types, where they (ii) act as master regulators of their targets, which are (iii) directly bound by these factors through specific *cis*-regulatory motifs.

Due to these characteristics, the regulatory action of CTS factors over their targets is usually assumed to be qualitative (on/off): if the master CTS factor is expressed, their direct targets will also be expressed in that specific cell type (Fig. 1B). This leads to CTS target programs whose expression or inclusion (for genes or exons, respectively) could be predicted solely by the presence/absence of the master CTS factor. However, each target gene or exon has a distinct sensitivity to its regulator, which is at least in part defined by the regulator-target interaction (strength of the binding motif, position, structural sequence context, interplay with core transcription/splicing signals, etc.). This can lead to the activation of “nested” subsets of targets in different cell types, depending on the levels of expression of the master CTS factor: when the regulator is highly expressed, most targets would be present; on the other hand, low expression of the CTS factor would only activate the most sensitive ones (Fig. 1B,C). Thus, under this model, termed “nested programs” model, a master CTS factor would act as a dimmer switch, rather than as an on/off switch. We have recently described and demonstrated this model for the CTS SF *SRRM3* and its microexon targets in neurons (high expression of the SF) vs. in the endocrine pancreas (low expression of the SF) (Juan-Mateu et al, 2023); described in further detail below). In addition to this model, it is also well known that different CTS (and general) factors can interact in a cell type-specific manner, giving rise to non-overlapping CTS target programs (e.g. Saito et al, 2019). In two different cell types, both models can be combined, resulting in more complex outcomes (Fig. 1B,D).

## Function and regulation of AS

Almost 50 years after the first evidences of “genes in pieces” (Gilbert, 1978; Berget et al, 1977), we know that most genes of multicellular organisms are split into sequences that form the mature mRNA (exons), separated by longer intervening sequences (introns), and that both exons and introns are transcribed into pre-mRNA molecules. The removal of introns and ligation of exons in the order they appear in a gene is a precise and efficient process known as splicing. AS represents a deviation from this canonical arrangement, where certain exons are skipped or included in a regulated manner resulting in various forms of mature mRNA. Thus, it can be considered that, while transcriptional regulation leads mainly to a quantitative regulation of the transcriptomes (how much a gene is expressed), AS leads to a qualitative one (which isoforms of a gene are expressed) (Mantica and Irimia, 2022). Although there is a long, ongoing debate on how much of the transcriptomic complexity produced by AS is reflected at the protein level (Blencowe, 2017; Weatheritt et al, 2016; Tress et al, 2017), recent proteomic studies show that a substantial fraction of the RNA isoforms are translated (Sinitcyn et al, 2023), and hundreds of single-case studies attest to the functionality of many AS events in different cellular contexts (Kalsotra and Cooper, 2011; Vuong et al, 2016; Kelemen et al, 2013). These functions range from changes in molecular properties of mRNAs and proteins (subcellular location, stability, DNA/RNA/protein binding specificity and affinity, etc.) to the modulation of nearly every possible cell behavior (proliferation, migration, adhesion, polarity, membrane depolarization, etc.).

Nearly half of the human protein-coding genes express multiple splice variants that are highly regulated across cell and tissue types. The vertebrate brain exhibits the highest prevalence of CTS AS, followed by muscle, while other tissues show certain distinct conserved AS profiles (Tapial et al, 2017; Fagnani et al, 2007). As mentioned above, these neuronal-specific AS patterns are generated according to their specific SRSs, in which CTS factors play a prominent role. In the next section, we will discuss the case of neural microexons and their master regulators, SRRM3 and SRRM4 (collectively referred to as Srrm3/4), as a paradigmatic example of this type of CTS splicing regulation.

## A paradigmatic CTS program: Srrm3/4-regulated neuronal microexons

By analyzing CTS AS, two studies (Irimia et al, 2014; Li et al, 2015) uncovered the existence of hundreds of very short exons, or microexons, whose inclusion was observed only, or mostly, in neural tissues, representing a remarkable example of a CTS splicing program. While any definition based on length is to some extent arbitrary, and other authors have considered different cut-offs (e.g. ≤30 nts (Liu et al, 2023; Volfovsky et al, 2003), or ≤51 nts (Li et al, 2015; Gohr et al, 2023)), we followed here the definition of our original publication: exons of length 3-27 nts (Irimia et al, 2014). Although these are only ~1% of all AS events, microexons were found to be the most conserved type of AS in vertebrates, comprising one-third of all neural regulated exons conserved between humans and mice (Irimia et al, 2014). Remarkably, some of them span 450 million years of evolution, being conserved from shark to human or even more distantly related species (Irimia et al, 2014; Torres-Méndez et al, 2022).

Given their very short length, microexon regulation is mechanistically challenging. The main advance in understanding their global regulation was the discovery that most neural microexons respond to the overexpression of *SRRM4* (Ser/Arg repeat-related protein of 100 kDa, also known as nSR100) in any non-neural context (Irimia et al, 2014; Raj et al, 2014). The regulatory effect of SRRM4 is usually dependent on the presence of a UGC motif next to the 3′ splice site of the upstream intron (Nakano et al, 2012; Irimia et al, 2014; Raj et al, 2014), but little else is known about other cis-regulatory requirements. Other SFs, such as the widely expressed SRSF11 and RNPS1 proteins, have been shown to act as positive co-factors for subsets of neural microexons (Gonatopoulos-Pournatzis et al, 2018). On the other hand, PTBP1 was found to antagonize the function of SRRM4 for a large fraction of microexons (Raj et al, 2014), and the core SF U2AF1 also has a conserved and somewhat unexpected role in repressing neural microexon inclusion (Torres-Méndez et al, 2022).

The functional importance of microexons is attested by multiple single-case studies (Quesnel-Vallières et al, 2015; Parras et al, 2018; Laurent et al, 2015; Ohnishi et al, 2014; Lewis et al, 2017; Gonatopoulos-Pournatzis et al, 2020; Gonatopoulos-Pournatzis and Blencowe, 2020; Poliński et al, 2023), as well as by the numerous neurodevelopmental defects of *Srrm4* mutant mice. Homozygous *Srrm4* mutant mice die after birth and undergo dramatic CNS alterations (Quesnel-Vallières et al, 2015), while heterozygous mutant mice have altered synaptic transmission and neuronal excitability resulting in autism-like behavior (Quesnel-Vallières et al, 2016). Also, a different (hypomorphic) mutation in *Srrm4* has been linked to deafness ((Nakano et al, 2012), see below)).

Phylogenetic and syntenic analyses showed that, within vertebrates, *Srrm4* coexists with two paralogs resulting from the two rounds of whole genome duplication: *Srrm2* and *Srrm3* (Torres-Méndez et al, 2019). *Srrm2* is a broadly expressed and ancient general SF acting on both constitutive and alternative splicing (Blencowe et al, 1998; Grainger et al, 2009). However, *Srrm3* is also a neural-enriched SF that shares a C-terminal domain of 39 conserved amino acids with *Srrm4* (eMIC, enhancer of microexons), which is necessary and sufficient for the inclusion of most neural microexons by interacting with early spliceosomal components (Torres-Méndez et al, 2019). Despite regulating overlapping targets in vitro (Torres-Méndez et al, 2019; Nakano et al, 2019), a complex interplay and spatiotemporal expression of *Srrm3*/*4* determines differential, yet largely redundant, SRSs in different neurons. For instance, a mutation in *Srrm4* in mice leads to defective AS in hair cells and deafness, while AS profiles are preserved in the brain (Nakano et al, 2012), partly due to the developmental upregulation of *Srrm3* (Nakano et al, 2019). On the contrary, mice with loss of *Srrm3* expression have reduced lifespan, tremors and ataxia, together with AS alterations and degeneration of the cerebellum Purkinje cells, where *Srrm4* expression levels are low (Nakano et al, 2019). In zebrafish, *srrm3* mutation, but not *srrm4*, leads to impaired visual function, with a stronger phenotype in the double mutant fish (Ciampi et al, 2022). All these studies highlight an incomplete redundancy for *Srrm3/4* paralogs in vivo, as observed for many other CTS SF families (e.g. NOVA (Raj and Blencowe, 2015)).

## The neuronal SRS

As mentioned above, each cell expresses a combination of general and CTS SFs at certain levels. This combination of regulators defines the SRS of the cell and will determine the splicing outcome, i.e., the specific programs of isoforms that will be expressed and their relative levels. By expressing some of the important master regulators we will describe in this section, neuronal cells share similar SRSs and thus neuronal-specific splicing outcomes.

Together with the SRRM3/4 regulators mentioned above, various key CTS components of the neuronal SRS have been extensively investigated. The first one studied in detail was the brain-specific NOVA family (with two members in vertebrates, *Nova1* and *Nova2*, collectively referred to as *Nova1/2*) (Jensen et al, 2000). Since then, many other CTS SFs have been reported, including members of the RBFOX, ELAVL and PTBP families (Jin et al, 2003; Calarco et al, 2009; Zhu et al, 2006; Boutz et al, 2007; Makeyev et al, 2007). The importance of these SFs and their co-regulated target programs is attested by the severe phenotypes associated with their disruption in model organisms. For instance, *Nova1/2* double knockout mice are paralyzed and die immediately after birth (Ruggiu et al, 2009), and *Nova1* and *Nova2* single knockout mice show apoptosis in motor neurons, alteration of synaptic activity and of axon pathfinding (Jensen et al, 2000; Huang et al, 2005). *Rbfox1* knockout mice have been shown to be sensitive to seizure and display increased neuronal activity in the hippocampus, while *Rbfox2* knockout mice display alterations of Purkinje cells in the cerebellum (Gehman et al, 2011). *Elavl* neuron-specific paralogs are important for neuronal differentiation and neurite outgrowth (Akamatsu et al, 2005; Ince-Dunn et al, 2012). In differentiating neurons, *Ptbp1* is repressed by microRNA-124, leading to *Ptbp2* expression that is maintained in later neurogenesis and mature neurons (Boutz et al, 2007; Spellman et al, 2007), a crucial step to ensure the generation of protein isoforms critical for neuronal differentiation (Linares et al, 2015; Makeyev et al, 2007).

**Figure 2:**
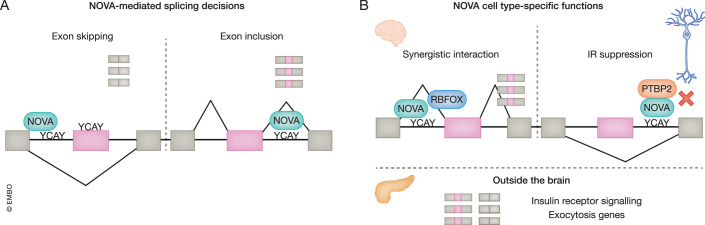
Nova as a paradigm of differential splicing decisions and cell type complexity. (**A**) Scheme of the NOVA1/2 positional dependency, where the binding to YCAY domains within the exon or in the upstream intron generally mediates exon skipping whereas binding to YCAY domains in downstream introns induces exon inclusion. (**B**) NOVA1/2 and RBFOX synergistic interaction (top left scheme); NOVA2 differentially regulates the same transcripts in excitatory vs inhibitory neurons. The figure shows that NOVA2 functions as a scaffold for PTBP2, impeding PTBP2-mediated Intron Retention (IR) in inhibitory neurons (top right scheme). Outside the brain, NOVA1 controls the AS of genes involved in insulin secretion and exocytosis in pancreatic beta cells (bottom scheme). Other examples include AS regulation by NOVA1 in adipose tissue (Vernia et al, 2016) and of NOVA2 in endothelium (Giampietro et al, 2015).

Despite the fact that these SF families can also shuttle between nucleus and cytoplasm to coordinate other layers of gene expression (Lee et al, 2016; Eom et al, 2013; Zheng et al, 2012; Linares et al, 2015), transcriptomic profiling of all these experimental models revealed programs of specific target exons assembled into biologically coherent networks (or regulons, (Keene, 2007)) that correlate well with the observed phenotypes (e.g. genes involved in synapsis and vesicle transport). Interestingly, these studies and other transcriptome-wide analyses of protein-RNA binding have shown a strong positional regulatory effect on the inclusion of the targets, dependent on the position of SF binding. For instance, RBFOX1/2/3 bind to UGCAUG motifs in pre-mRNAs (Jin et al, 2003; Ponthier et al, 2006). In general, if they bind in the downstream intron they enhance inclusion of the target exon, and if they bind in the upstream intron or in the target exon they inhibit inclusion. This positional dependency, or splicing regulatory map (Witten and Ule, 2011), was first discovered for NOVA1/2, which binds to clusters of YCAY motifs (Ule et al, 2006; Licatalosi et al, 2008), and has since been shown for most CTS SFs (Fig. 2A). Nevertheless, there are exceptions to this general rule, as certain SFs almost exclusively function as positive regulators of exon inclusion, such as SRRM3/4 (Irimia et al, 2014), or as negative regulators, such as PTBP1 (Hu et al, 2018), both of which tend to bind upstream of their target exons.

These studies indicate a broadly shared neuronal SRS. However, while this scenario is largely valid for most neurons and most CTS exons, this SRS can be further elaborated and diversified between neuronal subtypes. For instance, in some cases, the same CTS SF can exert different actions on the same transcript depending on the cellular context. NOVA2 provides again a paradigmatic example of this complexity (Fig. 2B). NOVA2 has distinct binding sites on the same transcripts depending on whether they are expressed in inhibitory or excitatory neurons (Saito et al, 2019). This occurs because NOVA2 works as a *cis*-acting factor for PTBP2, sequestering it and preventing PTBP2-mediated neuronal-specific intron retention only in certain neuronal subtypes, thus diversifying their AS programs (Fig. 2B). NOVA1/2 and RBFOX also show a similar positional dependency and they can either interact synergistically or antagonistically across the brain (Zhang et al, 2010) (Fig. 2B). In addition, specific CTS factors can be recruited in a highly neuronal subtype-specific manner. For example, *Khdrbs3*, also known as *Slm2*, has been shown to be essential for the functional specification of glutamatergic neurons in the hippocampus through the regulation of very few targets, mainly consisting of exons from synaptic proteins (Traunmüller et al, 2016). Among those proteins are the neurexins, which express different isoforms in glutamatergic and parvalbumin-positive interneurons due to the presence and absence, respectively, of *Slm2 (*Nguyen et al, 2017*)*. Neurexin exons are also controlled by the two paralogs of *Slm2*, *Khdrbs1* and *Khdrbs2* (*SAM68* and *Slm1*, respectively), creating subtle, yet essential, differences among neuronal subtypes (Iijima et al, 2011, 2014). While these examples point towards complex elaborations of the basic neuronal SRS, it has so far been difficult to comprehensively study and compare AS patterns among most neuronal subtypes. However, developments in single-cell RNA-seq (scRNA-seq) technologies are expected to soon shed light on how the neuronal SRS can be subtly modified to achieve neuronal subtype-specific AS programs [Box 1].

Interestingly, in addition to these, arguably mild, modifications of the basic neuronal SRS, it has already been reported that sensory neurons exhibit much more modified versions of this SRS (Ling et al, 2020). The best characterized case among them is that of the retina photoreceptors, which make use of a unique SRS of shared and distinct SFs to generate a transcriptome that is different from that of all other neurons. In the next sections, we will focus on photoreceptors as a noteworthy example to explore the diversification of SRSs.

Box 1 Approaches to detect variations of AS programs and SRSs across neuronal subtypes The brain encompasses hundreds of functionally and morphologically different cell types, making it intriguing to explore whether specific cell populations show more elaborate iterations of the canonical neuronal SRS leading to particular developmental and spatiotemporal AS differences. A few years ago, new approaches enabled the analysis of splicing patterns of sorted neuronal populations, revealing differences between neuronal subtypes (Zhang et al, 2014; Huntley et al, 2020; Nguyen et al, 2017). Concomitantly, various studies successfully applied scRNA-seq to profile neurons from specific brain regions and greatly improved our understanding of neuron subtypes at the gene expression level (Zeisel et al, 2015; Mardinly et al, 2016; Tasic et al, 2016; Paul et al, 2017; Hrvatin et al, 2018). Despite the fact that tools such as Smart-seq2 (Picelli et al, 2014), FLASH-seq (Hahaut et al, 2022) and VASA-seq (Salmen et al, 2022) allow AS analysis from scRNA-seq data, challenges remain in comprehensively capturing alternative isoforms, especially for lowly expressed genes. Current efforts, especially those employing long-read sequencing techniques, are starting to enable exploring the dynamics of neuronal CTS AS in single cells (Feng et al, 2021; Song et al, 2017; Karlsson and Linnarsson, 2017; Joglekar et al, 2021; Gupta et al, 2018; Joglekar et al, 2023; Parada et al, 2021).

## AS in retinal photoreceptors

**Figure 3:**
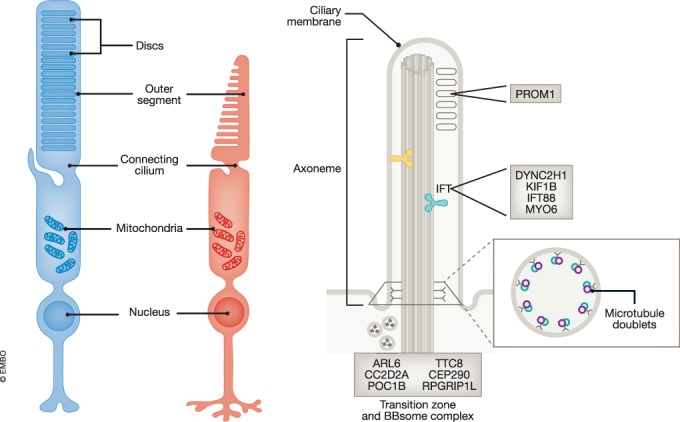
Photoreceptor and OS structure. Schematic representation of rods and cones (left) and higher magnification of the OS axoneme (right). Opsin-vesicles move from the Golgi to the basal body where they dock to Dynein (depicted in yellow) and get transported along the axoneme and delivered to the discs (depicted as gray circles). Upon cargo release, intraflagellar transport (IFT) components go back to the basal body through retrograde IFT, mediated by the Kinesin family (depicted in light blue). Many OS proteins (gray boxes) incorporate retina-specific exons (Ciampi et al and Murphy et al).

Photoreceptors are the light-sensing cells of the retina and include two types: cones and rods (Fig. 3). They are highly modified neurons, which have been morphologically and functionally specialized to receive and transduce light signals [Box 2]. In vertebrates, this specialization involves the Outer Segment (OS), a ciliated structure filled with membranous discs and adapted for high fidelity-photon capture, with strong metabolic requirements. The extreme cellular specialization of the photoreceptors is mirrored by their complex transcriptomes and proteomes, both at the gene expression and AS levels (Weyn-Vanhentenryck et al, 2018; Ciampi et al, 2022; Murphy et al, 2016; Hoshino et al, 2017).

During vertebrate eye development, AS is highly dynamic and several studies have shown that both human and murine retina exhibit various temporal changes in AS patterns that impact different sets of genes. One of the first studies comprehensively characterizing splicing in the murine retina discovered around 7000 exons showing temporal changes, with differential AS events occurring more frequently in early development (Wan et al, 2011). Two subsequent studies reported that genes involved in cilium and OS biogenesis and assembly, as well as genes from the centrosome, are highly alternatively spliced in early eye development (Hoshino et al, 2017; Mellough et al, 2019). At intermediate developmental windows, some SFs are themselves differentially spliced, suggesting that a new SRS is established to sustain further developmental AS changes. Finally, transition to the adult retina is associated with differential AS of genes encoding histone modifiers, as well as those involved in cilia, Golgi vesicle transport, axon and synapse formation (Mellough et al, 2019). These data suggest that a precise temporal production of isoform programs during photoreceptor development is instrumental for their maintenance and functionality. This is particularly extensive for OS genes, underscoring the contribution of specific isoforms to enable a highly specialized primary cilium in photoreceptors.

Importantly, many of these isoforms are produced by incorporating exons whose inclusion is specific to retinal transcripts (Ling et al, 2020; Murphy et al, 2016). For instance, we recently reported that the retina expresses the largest number of tissue-enriched microexons in all studied vertebrate species, and we identified a program of retina-specific microexons (RetMICs) that are highly evolutionarily conserved and enriched in photoreceptors (Ciampi et al, 2022). Genes harboring RetMICs are involved in vesicle transport along the microtubule axoneme and cilia biogenesis and function (green boxes in Fig. 3), and, for a few cases, clear biochemical differences between the RetMIC inclusion and skipping protein isoforms have been reported. For example, a RetMIC in *DYNC2H1* weakens microtubule binding, accelerating protein movement toward the OS tip (Niekamp et al, 2019). Similarly, a RetMIC in *KIF1B* promotes higher ATPase hydrolysis rate and motor activity (Matsushita et al, 2009). Moreover, RetMIC-containing genes are enriched among loci genetically associated with different human retinopathies. Consistent with these functional enrichments, a zebrafish mutant for *srrm3*, showing a large mis-regulation of RetMICs, exhibited severe OS alteration, photoreceptor death and blindness (Ciampi et al, 2022). However, despite the strong phenotype of a global microexon mis-regulation, it should be noted that depletion of individual RetMICs in mice have not shown any evident visual phenotypes (Matalkah et al, 2022), likely as a result of compensatory effects and the high robustness of living systems to relatively subtle perturbations as those exerted by individual microexon deletions.

All together, these findings indicate that photoreceptors harbor a unique proteome specialization by microexons that is necessary for the proper development and functioning of the OS. Indeed, in this structure, there is a uniquely high demand for vesicle formation, transport, and recycling (Khanna and Rao, 2015). RetMICs, alongside longer retina-specific exons, may aid in meeting this need by promoting protein interactions with specific substrates and enabling unique functional properties.

Box 2 Morphological and functional features of photoreceptors Rods and cones rely on the expression of different visual pigments (or opsin molecules), belonging to family 1 of G-protein coupled receptors. Rods have extraordinary sensitivity to light and can detect even a single photon (Baylor et al, 1979), while cones are 100 times less sensitive than rods but are faster during phototransduction and are responsible for bright-light vision. Structurally, each photoreceptor cell bears a single Outer Segment (OS), a ciliated structure filled with membranous discs and adapted for high fidelity-photon capture. This ciliary function is unique to photoreceptors and it is reflected by unique morphological characteristics. The OS has a microtubule axoneme of nine microtubule doublets (typically referred as 9 + 0 conformation), as found in all primary non-motile cilia. The numerous proteins involved in phototransduction are synthesized in the photoreceptor Inner Segment and then concentrated in the OS within its densely packed and organized membranous discs. Some of those proteins move extensively between the segments in response to light. Specialized mechanisms for protein trafficking are required to meet the extraordinary demand for new discs and transport of OS proteins. The axoneme is a crucial docking point for transport of motor proteins carrying vesicular cargo (Fig. 3). While multiple mechanisms, including random diffusion and binding of soluble proteins, are likely to play a role, intraflagellar transport (IFT) is arguably the most important for photoreceptors (Rosenbaum et al, 1999; Marszalek et al, 2000; Pazour et al, 2002) and different complexes, including the BBsome, are working at the ciliary transition zone to ensure its proper functioning.

## Assembling the unique SRS of photoreceptors

These studies revealed that photoreceptors exhibit very complex AS patterns, including both cell type-specific long exons and microexons that are not present in other neurons and non-neural cells. How has the SRS of photoreceptors diverged from that of other neurons to generate these unique AS programs? Various studies have shown that this is largely due to several key CTS that have different expressions in the two neuronal groups (quantitatively or qualitatively). Here, we review evidence pointing at the need for both the presence of specific CTS factors (positive regulators, e.g., MSI1, SRRM3) as well as the absence of key neural-specific ones (negative regulators, e.g. NOVA, RBFOX, ELAVL).

### Presence of positive regulators

The patterns of AS in retina and photoreceptors are known to be in part driven by the direct action of an RNA-binding protein called Musashi-1 (MSI1) (Murphy et al, 2016; Ling et al, 2020; Matalkah et al, 2022). The Musashi family comprises two members, *MSI1* and *MSI2*, and it has been involved in stem cell renewal and negative regulation of cell differentiation. Although it was known that *MSI1* has essential functions for vision (Susaki et al, 2009), the first evidence of its involvement in AS was given by Murphy and colleagues in 2016. They described a set of exons enriched in the mouse retina, specifically in post-mitotic photoreceptors, which was dependent on MSI1 (Murphy et al, 2016). Moreover, *MSI1* overexpression in neuroblastoma cells was shown to promote the inclusion of some of these photoreceptor-specific exons by binding to the downstream proximal introns. *Msi1* and *Msi2* have a distinct pattern of expression across retinal development, with *Msi1* being highly expressed at birth until postnatal day 16, while both proteins show equal expression in adult photoreceptors (Matalkah et al, 2022). Indeed, both genes are functionally redundant in mature retina, as inducible double knockout mice have a strong visual impairment and mis-splicing of photoreceptor exons, while single-knockout mice have no alterations in photoreceptors (Matalkah et al, 2022). On the other hand, single-knockouts at earlier stages show developmental phenotypes, particularly for *Msi1*, due to the low expression of *Msi2* at those time points (Sundar et al, 2021). In a study from a different group, the analysis of the expression of various SFs across numerous mouse cell types allowed for the identification of MSI1 and PCBP2 as candidates for inducing rod-specific splicing patterns (Ling et al, 2020). After robust overexpression of *MSI1* or *PCBP2* (with or without simultaneous *PTBP1* knockdown) in liver cancer cell lines, Ling and colleagues observed that *MSI1* expression combined with downregulation of *PTBP1* is linked to the activation of photoreceptor-specific exons. Finally, we also observed that MSI1 can induce the upregulation of certain retinal exons in HEK293 cells, primarily favoring longer exons rather than microexons (Ciampi et al, 2022).

**Figure 4:**
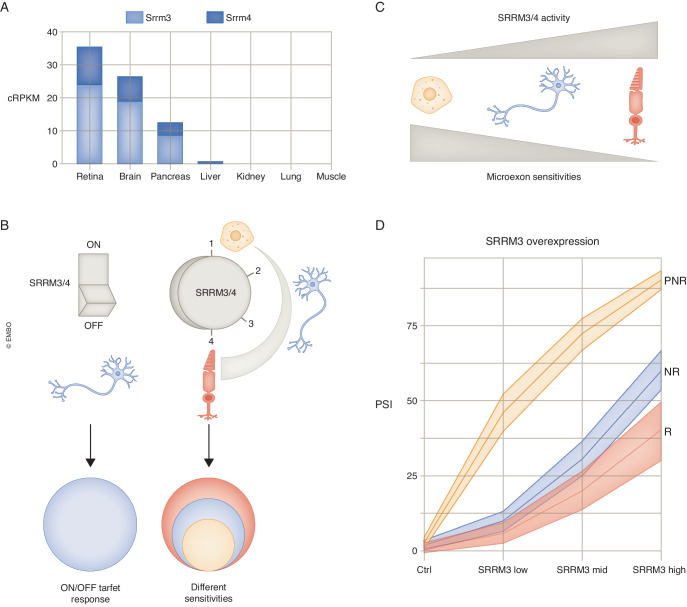
A new paradigm for microexon regulation in different cell types. (**A**) Different tiers of *Srrm3/4* expression (cRPKM) are observed in mouse retina, brain and pancreatic islets (data from *VastDB (*Tapial et al, 2017*)* and (Juan-Mateu et al, 2023)). (**B**) Summary of the proposed regulatory model for CTS microexons. On the left, the classical on/off switch generates a single CTSprogram. On the right, distinct tiers of expression of the master regulators in different cell types activate increasingly larger sets of targets due to their intrinsic sensitivity to the regulator, generating a triple-nested program. (**C**) Microexon sensitivity in pancreatic cells, neurons and photoreceptors is inversely proportional to the activity of the master regulators. (**D**) Microexon inclusion (PSI) upon different *SRRM3* overexpression levels in HeLa cells (data from (Juan-Mateu et al, 2023)). PNR: subprogram shared by all three tissues, IsletMICs from (Juan-Mateu et al, 2023); NR: subprogram for neural and retina only, from (Juan-Mateu et al, 2023); R: Retina-only subprogram, RetMICs from (Ciampi et al, 2022).

A second important CTS SF, specifically for the inclusion of RetMICs and neuronal microexons and other short exons in photoreceptors, is SRRM3. In vitro, most RetMICs do not require MSI1, and its overexpression in HEK293 cells leads to the inclusion of only a handful of microexons (Ciampi et al, 2022). In contrast, the inclusion of most RetMICs, as well as that of neural microexons, can be largely promoted by either SRRM3 or SRRM4 alone. Overall, evidence from different studies suggests that MSI1 is not sufficient for RetMIC inclusion but may be necessary in vivo, while SRRM3/4 are necessary and sufficient, both in vivo and in vitro (see also below). However, *Srrm3* expression increases during photoreceptor development, while *Srrm4* levels progressively go down. Consistently, in vivo deletion of *srrm3*, but not of *srrm4*, led to photoreceptor and vision phenotypes in zebrafish. Interestingly, the combined expression of *Srrm3/4* seems particularly high in the retina, even compared to other neural tissues (Fig. 4A). A recent study showed that different levels of *Srrm3/4* are sufficient to generate two nested programs of CTS microexons in endocrine pancreas (which has low but substantial *Srrm3* expression) and neurons (high *Srrm3* and *Srrm4* expression) (Juan-Mateu et al, 2023). This nested pattern was largely driven by the differential sensitivity of microexons to the action of SRRM3/4: microexons shared by endocrine pancreas, neurons and retina (PNR program) have much higher sensitivity than neural and retina ones (NR program). Thus, the low levels of *Srrm3* in pancreas were sufficient to drive the inclusion of PNR microexons, but not of NR microexons. Hence, these findings indicate that, rather than adhering to the conventional “on/off” model of master regulators (as originally proposed, and presented in the section “A paradigmatic CTS program: Srrm3/4-regulated neuronal microexons” above), this nested regulatory model suggests a more complex “multi-level switch” paradigm. According to this paradigm, increasing expression levels of a master regulator in different cell types activates progressively larger sets of its targets based on their inherent sensitivity to the regulator (Fig. 4B). Importantly, both PNR and NR microexons are included in the larger program of microexons observed in the retina, configuring a triple-nested program (Fig. 4B). This suggests that RetMICs (referred as “R” here), which are unique to the retina, could correspond to microexons with very low sensitivity to SRRM3 (Fig. 4C). In line with this hypothesis, a re-analysis of RNA-seq data for HeLa cells expressing increasing levels of *SRRM3* from (Juan-Mateu et al, 2023) shows that only PNR microexons respond strongly to the lowest level of SRRM3 activity, whereas NR microexons and, especially, the RetMICs (R) require much higher SRRM3 activity for inclusion (Fig. 4D). It is worth noting that this nested model can contribute to further elaborate and diversify any Regulatory State, as it could be applied to other neural master regulators -either SFs and TFs- that exhibit low expression levels in cell types beyond the CNS (e.g. NOVA1 in brain versus pancreas (Villate et al, 2014), NOVA2 in brain versus endothelial cells (Giampietro et al, 2015) or POU3F3 in brain vs kidney (Nakai et al, 2003)).

### Absence of negative regulators

**Figure 5:**
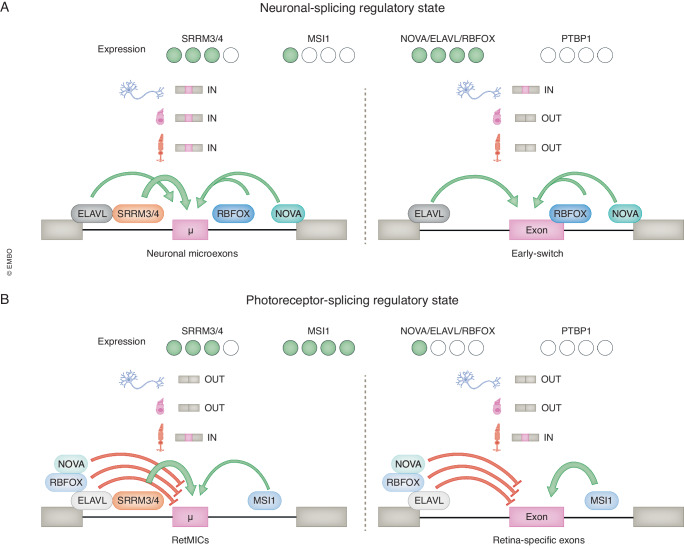
Schematic representation of Neuronal- and Photoreceptor-SRS. For each SRS, the SFs expressed in either non-sensory neurons (**A**) or photoreceptors (**B**) are shown, depicted as green (expressed) or white (not expressed) ellypses, depending on the expression level. The drawing below represents their differential binding to upstream and downstream introns leading to exon inclusion (IN) or skipping (OUT). High levels of *SRRM3/4* expression lead to inclusion of neuronal microexons (µ) in both non-sensory (upper cell cartoon), photoreceptors (bottom cartoon) and other sensory neurons (middle cartoon). In contrast, only non-sensory neurons include early-switch neural exons, as they require the positive effect of RBFOX, NOVA and ELAVL, not sufficiently expressed in photoreceptors and other sensory neurons. Finally, the lack of those negative regulators, together with the positive action of the master regulators SRRM3/4 and MSI1, is likely to promote inclusion of photoreceptor-specific microexons (RetMICs) and longer exons. Photoreceptor longer exons rely more on MSI1 activity rather than SRRM3/4, while the converse is true for RetMICs.

Another important component of the photoreceptor SRS seems to be the intriguing absence of crucial neuronal SFs. For instance, although the low sensitivity of RetMICs together with particularly high levels of *Srrm3/4* is likely to partly contribute to the generation of a unique SRS for microexons in photoreceptors, it is not sufficient to explain why neurons, which also have high levels of *Srrm3/4*, do not include some RetMICs at all (Ciampi et al, 2022). Moreover, as also mentioned above, beyond microexons, the splicing programs of photoreceptors and other neurons are quite divergent. A study from Weyn-Vanhentenryck and colleagues was in fact the first to report that sensory neurons, including photoreceptors, have a distinct developmental splicing program compared to other CNS neurons (Weyn-Vanhentenryck et al, 2018). In this study, they derived alternative exon switches matching neuronal developmental stages and defined early-switching and late-switching exons (mainly referring to “canonical” long exons). Unexpectedly, sensory neurons preserve an immature-like splicing pattern. In particular, these cells lack early splicing switches, retaining a splicing profile reminiscent of immature CNS neurons, yet they show a relatively mature splicing pattern for late-switch exons. This seems to be due to the fact that sensory cells, including photoreceptors, have no expression of the pan-neuronal regulators *NOVA* and *RBFOX,* which hampers the promotion of early-switch exons. This observation has been confirmed by other studies, including ours, showing that photoreceptors have very low expression of key neural SFs (Ciampi et al, 2022; Murphy et al, 2016). As discussed above, these factors are major components of the non-sensory neuronal SRS, as they promote the inclusion of the canonical CTS exons and microexons in non-sensory neurons (**Neuronal-SRS**, Fig. 5A). Another important absence, in this case shared among all neuronal types, is *PTBP1*, whose expression steadily decreases upon neuronal differentiation.

While the absence of these neural factors seems to play a role in defining the unique SRS of photoreceptors by not enabling the inclusion of early-switch exons, it is possible that they are also important to ensure RetMICs are only included in photoreceptors by repressing their inclusion in other neurons. We found a significant enrichment for known binding motifs for NOVA and RBFOX in the upstream introns of RetMICs (Ciampi et al, 2022), where their binding is expected to cause exon downregulation (Witten and Ule, 2011). This was in contrast with neural microexons, which showed enrichment for inclusion-enhancing NOVA and RBFOX binding motifs in the downstream introns, as previously reported (Li et al, 2015; Matsushita et al, 2009; Murphy et al, 2016). ELAVL, which does not exhibit such conserved positional dependency, binds upstream in both neural and RetMICs. Therefore, we propose that these factors (NOVA, RBFOX, ELAVL) exert a positive effect on neural microexons in non-sensory neurons alongside with SRRM3/4 (Fig. 5A), but a negative effect on RetMICs in those cells (Fig. 5B). On the other hand, the lack of repression of RetMICs by these neural factors, together with the positive effects of high levels of SRRM3/4 and MSI proteins, can be conceived as the basis for the Photoreceptor-SRS (Fig. 5B). While this model is centered around microexons, it is plausible that it also applies to retina-specific longer exons, which are more responsive to MSI1 rather than SRRM3/4, and might similarly be repressed by NOVA/RBFOX/ELAVL in non-sensory neurons (Fig. 5B). Importantly, it should be acknowledged that this is a proposed model that remains to be experimentally tested and may not apply to all events within each (micro)exon category.

## AS and SRS in other ciliated sensory cells: hair cells and olfactory neurons

In addition to retina photoreceptors, a variety of other cell types are appointed to sensory stimuli perception in vertebrates. To what extent do they share similar AS profiles and underlying SRSs with photoreceptors and/or non-sensory neurons? Although the information is very scarce, we here focus on hair cells and olfactory neurons which, similarly to photoreceptors, have distinct specialized ciliary structures whose malformation due to mutations result in human diseases.

In contrast to the photoreceptor OS, inner and outer hair cells bear dozens of F-actin microvilli, known as stereocilia, which are organized in a stair-like fashion to maximize their deflection. Those of the shorter rows have mechanosensitive channels at their tips (Beurg et al, 2009) that open upon sound-induced vibration, allowing cell depolarization and the release of neurotransmitters onto nerve fibers that carry the auditory information to the CNS (Zheng and Holt, 2021). In contrast to other actin structures, mature stereocilia actin turnover is constrained to the tips with little incorporation along the length, creating a very stable core, most likely to preserve stereocilia length and longevity (Narayanan et al, 2015).

The first SF to be associated with hearing was, in fact, the master regulator of microexons *Srrm4*, which was found mutated in Bronx Waltzer mice (Nakano et al, 2012). Similarly to photoreceptors and other neurons, SRRM4 targets in hair cells comprise stereocilia genes as well as secretory and synaptic genes. Since then, other SFs have been identified as relevant for hearing and hair cell development and function (Shi et al, 2022). *Sfswap* is widely expressed in mice developing inner ear and mature cochlea (Moayedi et al, 2014). The functional loss of *Sfswap* has been linked to impaired inner ear patterning, characterized by a decrease in the number of outer hair cells and the presence of ectopic inner hair cells in the cochlea (Moayedi et al, 2014). However, the target exons of *Sfswap* and the specific mechanisms by which it regulates inner ear-specific AS remain unclear. Whole-exome sequencing to investigate the genetic basis of nonsyndromic hearing loss revealed pathogenic mutations in the SF *ESRP1*, which led to abnormal splicing of genes involved in cochlear development (Rohacek et al, 2017). Subsequent analysis using patient-derived induced pluripotent stem cells and animal models provided additional evidence for impaired inner ear morphogenesis and hair cell function in *ESRP1* mutants (Rohacek et al, 2017). Interestingly, *ESRP1*’s role in inner ear development is likely conserved in zebrafish, as mutation of its ortholog together with *esrp2*, leads to severe malformations of this structure (Burguera et al, 2017). Finally, deletion of *Rbm24* in mice has been found to impact the inclusion of *Cdh23* exon 68, specific to the inner ear. This led to hearing loss and impaired motor coordination (Zheng et al, 2021). CDH23 and CDH15 are essential components of the tip links, which connect the mechanosensory stereocilia and the kinocilium in the hair bundle, enabling mechanotransduction (Richardson and Petit, 2019). Similarly, zebrafish *rbm24a* mutants showed structural alterations of stereocilia and kinocilia in hair cells as well as impaired hair cell activity (Ghilardi et al, 2021).

Olfactory cilia, projecting from the olfactory epithelium within the mucus, harbor a microtubule-based axoneme common to motile cilia but they lack the dynein arm necessary for movement and thus are immotile (Menco, 1984). Olfaction signaling is mediated by odorant ligands that bind to G protein-coupled receptors, a single type for each olfactory neuron (Niimura and Nei, 2003). The odorant binding generates an increase in cAMP, opening of ion channels, and depolarization of the neurons which is amplified via a Ca2+-activated chloride channel and transmitted to the brain, causing the sensation of smell (Patel and Pinto, 2014; Berbari et al, 2009).

Studies about AS in olfactory neurons are even more sparse than for photoreceptors and hair cells. Two decades ago, the existence of some alternative exons was reported for olfactory receptors (Young et al, 2003), but no cell type specific isoforms or SFs have so far been described in vertebrates. A recent work in *C. elegans* identified a short AS isoform of *mec-2A*, preferentially expressed in olfactory neurons and regulated by the *SF mec-8*, as crucial for olfaction (Liang et al, 2021).

**Figure 6:**
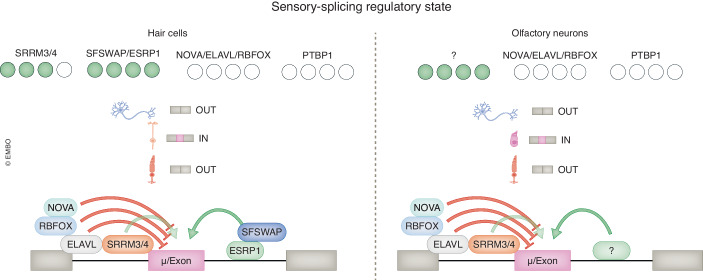
Schematic representation of the Sensory-SRS. Hair cells and olfactory neurons have low expression of *RBFOX*, *NOVA* and *ELAVL*, which likely act as negative regulators as per photoreceptor-specific exons. This is accompanied by cell type-specific positive regulators, such as SRRM3/4, SFSWAP and ESRP1 in hair cells. The positive regulator(s) of the olfactory neurons are yet to be described.

As mentioned in the previous paragraph, sensory cells including hair cells and olfactory neurons show an immature-like splicing pattern characterized by limited expression of the neural SF families NOVA, RBFOX and ELAVL, and skipping of early-switch exons (Weyn-Vanhentenryck et al, 2018). In addition, their SRS is likely to be generated by some positive regulators, such as ESRP1 and SFSWAP for hair cells, promoting cell type-specific exon inclusion (Fig. 6). Further work will be needed to decipher the composition of these individual Sensory-SRSs.

## Future perspectives

Here, we have compared the AS regulatory profiles of sensory and non-sensory neurons using the SRS framework. We have focused on a few relevant CTS splicing regulators, which have been implicated in the regulation of CTS exons and microexons, particularly in neural cells. While these regulators have a strong impact in defining SRSs, we acknowledge that many other SFs, as well as some structural and epigenetic traits as well as gene transcription rates (mainly determined by Transcriptional Regulatory States), are likely to be important to define the complete SRS of each individual cell type. We propose that integrating their role within the SRS framework will help understanding how transcriptomes are ultimately generated. In addition, it is important to acknowledge that the SRS of a given cell type is dynamic, and thus can be modulated in time and space (e.g. by neuronal activity, cell-to-cell signaling, etc.).

Looking forward, a complete portrait of each cell type’s SRS will help unveiling splicing vulnerabilities. For instance, the second most common cause of autosomal dominant Retinitis Pigmentosa, accounting for 15–20% of all cases, is linked to mutations in the ubiquitously expressed pre-mRNA processing factor (*PRPF*) genes, encoding core components of the spliceosome (Wheway et al, 2020). Malfunctioning of these genes seems to be tolerated in most human tissues yet they are pathogenic in the retina, where photoreceptors are affected and degenerate. Although it remains unclear why mutations in core SFs affect only this cell type, and even though these are not always fully penetrant (e.g., due to differences in the Transcriptional Regulatory State (Rose et al, 2016; McLenachan et al, 2021)), it seems obvious that the explanation largely lies in the interplays within its unique SRS. A similar scenario can be envisioned for other sensory cells. For instance, in the case of hair cells, mutation of the SRRM4-dependent regulation of an exon in *REST* is responsible for hereditary deafness in human patients (Nakano et al, 2018) and mutations in the SF *ESRP1* are closely associated with sensorineural hearing loss in humans (Rohacek et al, 2017). In summary, characterizing the SRSs of each cell type will be an essential task to fully understand the impact of AS in both physiological and pathological conditions.

## Glossary

Master regulator: Protein (e.g. a TF or SF) that is at the top of a regulatory hierarchy, and whose activation is usually necessary and often sufficient to drive a specific cell fate by switching on the expression of a set of target genes/exons.
Syntenic analysis: Study of the conservation of gene order and arrangement in the genomes of different species.
Inner Segment: The portion of photoreceptors located between the OS and the cell nucleus. It contains various organelles and machinery necessary for the maintenance and energy production of the cell.
BBsome: Protein complex of eight subunits involved in sorting and trafficking proteins to and from the cilium.
Centrosome: Organelle that serves as the main microtubule-organizing center, involved in cell division and organization of the cytoskeleton.
Retinitis Pigmentosa: A group of inherited eye disorders characterized by the progressive degeneration of the retina. This condition typically starts with night blindness and gradually leads to a loss of peripheral vision, followed by a reduction in central vision, ultimately resulting in severe visual impairment or blindness.
Bronx Waltzer mice: Mouse strain carrying a mutation in a gene responsible for hearing and balance.
